# More Needs to Be Done During the Informed Consent Process

**DOI:** 10.7759/cureus.23346

**Published:** 2022-03-20

**Authors:** Rashed Alfuzaie

**Affiliations:** 1 Ophthalmology, Ibn Sina Hospital, Sabah Health Region, KWT

**Keywords:** retinal detachment, ethics, informed consent, retina, ophthalmology

## Abstract

Retinal detachment is an emergency commonly encountered in ophthalmic practice. In this article, a reflection about the ethical implications surrounding the informed consent process of retinal detachment repair is presented. We look at how premade informed consent forms allow for a better patient understanding of their condition, the procedure they are due to undergo, their postoperative course, and the potential complications they might face, hence potentially improving the overall outcome.

## Introduction

Retinal detachment is a serious condition whereby the inner neurosensory layer of the retina, which harbors the photoreceptors necessary for visual perception, separates from the retinal pigment epithelium. The incidence of this condition is ever increasing worldwide due to an aging population, diabetic retinopathy, myopia, and cataract surgery [1]. The degree of urgency to intervene varies from one case to another, owing to the presence or absence of macular involvement, which contains the highest density of cone photoreceptors necessary for color and central vision. Management does not solely rely on the surgery itself, but also on the patient’s understanding of the situation and their compliance to the surgeon’s instructions postoperatively. This can be potentially enhanced through proper education and an informed consent process.

## Case presentation

A 55-year-old expatriate male presented to an eye emergency department in Kuwait with painless visual loss in the left eye spanning one week. Past ocular history was significant for cataract surgery in the left eye done six years ago. Past medical history revealed aortic valve replacement through open-heart surgery performed seven years ago. At-home medications include warfarin and aspirin. Visual acuity unaided was 20/80 in the right eye and hand motion (HM) in the left eye. Vision improved in the right eye through pinhole apparatus to 20/20, but no change in the left one. Examination of the anterior segment has a posterior chamber intraocular lens (PCIOL) in the left eye and normal in the right. Posterior segment examination unfolded a pathologic myopic fundus in the right eye and a superior rhegmatogenous retinal detachment (RRD) involving the macula in the left with a superonasal horseshoe tear.

## Discussion

Patient’s perspective

Retinal detachment is an uncommon, yet established, complication after cataract surgery with an increasing risk each year [2]. Being a high myope also increases the risk owing to the increased axial length of the eye. The consequences being experienced now could have been from either mechanism. Neither was explained to him in the past. Today, the surgeon does not fully explain the disease or discuss management options with the patient. The only thing that was briefly communicated was his poor prognosis given the site of the detachment. Furthermore, the surgeon was not completely sure that a delay in management was warranted to prevent hemorrhage given his bleeding diathesis as his international normalized ratio (INR) was 4, which was outside the therapeutic range for warfarin patients. This was contrary to principles released by the Eye Physicians and Surgeons of Ontario (EPSO), which are one of the first ethical guidelines tailored to ophthalmology. The EPSO states that physicians are obliged to provide all information needed to the betterment of the decision-making process and answer questions raised by the patient to the best of their knowledge [3]. Since this was not an ordinary scenario, the EPSO also encourages asking for second opinions from colleagues and providing the support required to the patient in reaching a decision.

What evidence do we have?

Several papers were published proving that many macula-off cases can achieve a significant, although not perfect, restoration in vision [4-6]. The largest study on vitrectomies for patients on warfarin found a minimal increase in risk for hemorrhage postoperatively in the high INR group, and the overall trend in both groups was no drastic visual deterioration or need for further surgical intervention [7]. Looking at the corroboration, we can say that early intervention is warranted in this case.

Cultural context

The patient’s anticoagulant regimen was altered by the cardiology team in an effort to reduce the INR. Eventually, the surgeon decided to proceed with the surgery a week after the INR became 2.8, which is acceptable. However, after a brief explanation of the procedure, the patient initially declined the surgery. The problem stems from the fact that he was given a poor prognosis from the beginning and the idea of nothing improving became embedded in his head. Furthermore, while the surgeon explained some of the complications associated with the surgery, he did not cite references as to the chances of developing them. The patient was content with living with one eye, a common theme encountered in our daily practice, which might be attributed to hidden cultural factors. Eventually, the patient placed his full trust in the surgeon and agreed to undergo the retinal detachment surgery, although he did not completely understand the need for it or what was written in the informed consent form, another cultural factor that came into play, whereby surgeons hold a high social status and it would be wrongful to act as opposed to their recommendations. This creates a scenario whereby it is difficult to gauge the patient’s understanding, ideas, and expectations, all of which were not documented in his medical record. This raises several questions of whether we should reevaluate the consent process and how could we change it, and should we empower our patients’ knowledge with internationally referenced sources.

What can we do differently?

One way to address this issue is the utilization of premade consent forms approved by various consultants from reputable bodies, containing what is deemed to be reasonable for the patient’s understanding. A preprinted consent form offers numerous benefits with regard to improved readability as highlighted from a study done on orthopedic patients conducted by Owen et al. [8]. This can prove to be detrimental in dealing with illiteracy, which can be further complicated when consent is made with poor handwriting. It also allows for a more efficient process and saves the physician time.

Generic informed consent forms do not encourage the documentation of the patient’s ideas and expectations. This is an important yet often neglected aspect. A study done by Baker et al. at the Birmingham Midland Eye Centre found that preprinted forms allow better documentation of patients’ wishes when compared to handwritten ones [9]. The study also brings to light a problem that might be commonly disregarded related to our practice, being that a good portion of ophthalmology patients are unable to read due to poor vision. Hence, it is of utmost importance to read them out loud, as opposed to the case reported above. Perhaps soon, when designing a premade consent form, we bear this in mind and ensure the text is large enough to read or contains Braille prints for wider accessibility.

A preprinted consent form was evaluated by Krishnamoorthy et al. on cardiac surgery patients. Their study illustrated higher satisfaction rates following their surgeries as the spread of information to patients was uniform, allowing greater understanding and no discrepancy among physicians attending to their care [10]. Although one might argue that one form does not fit all, it might be more sensible to include some free space to add patient-specific information.

Professional guidelines

The American Academy of Ophthalmology (AAO) is renowned for being a pioneer in educational innovations. It is the go-to source for the learning ophthalmologist as it set the gold standards for our practices today. That being said, truly little variability in information sharing between physicians can be exhibited once we all approve their list of proposed complications. While designing a leaflet for such cases can be a mundane task, the AAO does provide leaflets available for purchase on their website [11,12]. This can prove to be a valuable source as the data is constantly updated and widely accessed by residents worldwide.

**Figure 1:**
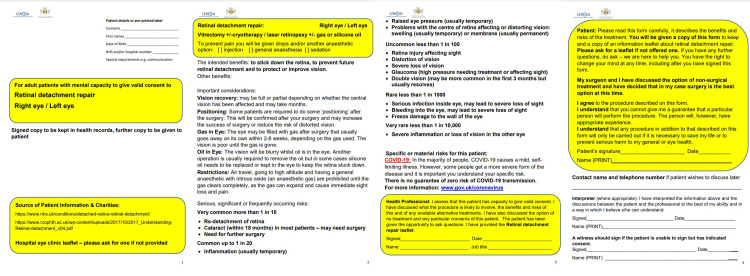
RCOphth and UKOA retinal detachment repair consent form

Through a collaborative effort done by the Royal College of Ophthalmologists (RCOphth) in London and the United Kingdom Ophthalmology Alliance (UKOA), a model premade consent form for retinal detachment surgery was made. It briefly explains the purpose of the procedure, important considerations to keep in mind, and the risks associated with the chances [13]. Figure 1 illustrates their model.

I believe should this patient had been provided with a handout explaining the disease, the outcomes of the surgery, the chances of success, and complications, they might have potentially altered their views and could promote better sense of self-care. For example, our patient could have been educated as to why surgery is detrimental to not only restore vision potentially but also prevent further complications down the line such as proliferative vitreoretinopathy (PVR). Some aspects that could have been discussed include the use of both endolaser and gas insufflation intraoperatively. While they can impair vision to an extent, their use is vital for fixing the retina in its place to prevent future adverse events, not to mention as to why positioning himself face down at home could impact the chance for a successful surgery and avoid complications such as gas cataracts.

One reported problem encountered in a study done by Courtney and Royle for inguinal hernia repair patients is the inconsistency in reported complications rates between hospitals in the United Kingdom. As demonstrated by their study, the quality of consent through premade forms was negatively impacted [14]. It can be difficult to agree upon complication rates between different centers due to a range of factors. These variations can be due to surgeon expertise, facilities available, and patient demographics.

An important topic worth mentioning is the lack of discussion with regard to ocular complications relating to local anesthesia as highlighted by the Royal College of Ophthalmologists (RCOphth) in London [15]. The generic consent form only covers the risks of general anesthesia, and the regular consent form has truly little space to add those few points. It can be difficult to justify why the surgeon switched from topical anesthetic drops to needle block. As this patient has suffered a subconjunctival hemorrhage in the postoperative period, which was likely due to the local injection received and his bleeding tendency, educating him prior to surgery might reduce any concern or anxiety that could be caused by this. Of note, physicians must not feel reluctant to include local anesthesia side effects owing to the fact of their infrequency.

Ethical principles

Autonomy is a cornerstone in medical ethics. Respecting the patient’s wishes not only builds a strong doctor-patient relationship but also empowers them to make health-related decisions. The Canadian Medical Education Directives For Specialists (CanMEDS) framework instated by the Royal College of Physicians and Surgeons of Canada (RCPSC) proposes that, for a physician to be competent, they must exercise proper informed consent process [16]. These are thoroughly discussed under the headings of “medical expert,” “communicator,” and “scholar.”

The College of Physicians and Surgeons of Ontario (CPSO) sets policies with regard to consent to treatment. Various points are present in the CPSO guidelines that could help us tailor the consent process based on the patient’s circumstances [17].

With regard to “general expectations,” it is essential to overcome communication barriers and use interpreters at times. Going back to our case, the patient was a middle-class labor worker with limited language skills. This raises several considerations. His level of education was not appraised prior to this, as was his understanding of the macula not being assessed. No analogies were used to explain his condition and surgery, and he was not offered an interpreter to aid his understanding and decision-making process.

On “obtaining consent,” the CPSO recommends that it should be voluntary without any coercion. Since the patient was told that his condition holds a poor prognosis, why did he proceed with the surgery in the first place since he was content with living with one eye? Could this have been due to pressure from the surgeon? Did he understand that there may be no need for urgent intervention? It is hard to tell as documentation was lacking.

This brings us to how the CPSO recapitulates the notion of documenting the details of informed consent process in the patient’s medical record. Should there be a problem faced, this can always serve as a testament for the physician and patient.

The CPSO guidelines also highlight the importance of a delegated physician, with the appropriate level of knowledge, to take the consent. In this case, it was a junior doctor. Could this have greatly impacted the patient’s understanding? Did the patient feel less satisfied with their explanation owing to being enlightened of their career status?

Another often neglected aspect is the lack of acknowledgment of the patient’s socioeconomic status. This goes against the AAO’s code of ethics, which strongly encourages examining the patient’s psychosocial status and occupational requirements prior to the commencement of treatment [18]. As he was an expatriate, the idea of living with one eye seemed more feasible than the costs incurred from the surgery and its chances of poor outcome.

Reflection and take-home messages

Days after the INR normalizes, the patient undergoes pars plana vitrectomy (PPV), gas insufflation, and endolaser surgery. Four months after his presentation, the patient’s visual acuity reaches 20/25 in the left eye, and the retina is flat. Much against expectations, the patient made a marked recovery. This highlights the importance of the notion of beneficence, in which we as doctors have to always work in the patient’s best interest. This is done through thorough researching of the disease and the impacts of various factors on a successful surgery, delivering accurate information to the patient and explaining their disease, and carefully planning the management with them.

## Conclusions

The ophthalmology scene is flourishing worldwide. Several eye institutes are evolving to achieve international recognition from professional bodies. One way to go about this is improving the ethical practices and informed consent process. Looking at the previously reported case, there was a deficit in the patient’s understanding of the situation and potentially in the informed consent process. This is a recurring theme not only in ophthalmology but also in other specialties. There are numerous learning points that can be derived from this experience. In order to deliver the best care possible, it is advisable to follow recommendations set by international bodies. This include amending laws, governing ethical practice, and using premade leaflets and consent forms tailored to the disease and procedure required. This helps tackle any uncertainty patients and physicians might face in their daily practice. More healthcare bodies should adopt the use of premade consent forms and use information based on other experiences from various institutions.
